# Comparison of tension band wiring and other tibial tuberosity advancement techniques for cranial cruciate ligament repair: an experimental study

**DOI:** 10.1186/s13028-019-0481-1

**Published:** 2019-10-02

**Authors:** William McCartney, Ciprian Ober, Maria Benito, Bryan MacDonald

**Affiliations:** 1 NOAH, 38 Warrenhouse Road, Baldoyle, Dublin 13, Ireland; 2 School of Mechanical and Manufacturing Engineer, Dublin City University, Dublin, Ireland; 3 University of Agricultural Sciences and Veterinary Medicine, Calea Manastur 3-5, Cluj-Napoca, Romania; 4 Sandyford, Dublin 18, Ireland

**Keywords:** Cranial cruciate ligament rupture, Osteotomy, Tension band wiring, Stifle joint tibial tuberosity advancement

## Abstract

**Background:**

Cranial cruciate ligament (CCL) rupture is one of the most common causes of limb lameness in dogs. Surgical techniques based on tibial osteotomies such as tibial plateau leveling osteotomy and tibial tuberosity advancement are used to eliminate dynamic thrust. Tibial tuberosity advancement (TTA) uses an osteotomy fixated by cage, plates, forks and screws to change the relationship of the patellar tendon and tibial plateau angle. Tension band wiring technique is one of the most common surgical methods used to treat a tension fracture and remains the gold standard for the treatment of tibial tuberosity fractures. In this study, we compared experimentally the biomechanical effect of application of tension band wiring compared to other techniques for the fixation of the TTA osteotomy. The techniques compared to are standard commercially available systems for TTA fixation.

**Results:**

Tension band wiring (TBW) presented the higher resistance to failure compared to all the other surgical procedures, with the highest values found in the TBW group with 1.47 ± 0.07 N and the lowest in the TTA cage (0.82 ± 0.08) and TTA-2 (0.85 ± 0.06) groups with statistically significant differences in all cases (P < 0.001). TTA rapid and TTA plate groups exhibited a similar strength, and same happened between TTA-2 and TTA cage groups. All the other comparisons by pair were significantly different with P < 0.001.

**Conclusions:**

Results suggest that fixating the osteotomy with tension band wiring increases the strength of the fixation and decrease the risk of implant failure. Further clinical studies are needed to demonstrate in vivo reliability and to test different variables such as size and weight of dogs. These results could have important clinical implications in the treatment of CCL ruptures.

## Background

Cranial cruciate ligament rupture (CCL) is one of the most common cause of pelvic limb lameness in dogs [1–3]. In most cases, CCL rupture happens as a result of long-term degeneration and weaken of ligament over time due to genetic factors [3]—with certain breeds such as Labrador being predisposed [1–4]—obesity and certain joint inflammatory conditions may also play a role. CCL rupture is associated with meniscal damage and degenerative joint diseases [5].

The surgical techniques described to stabilize the unstable stifle joint in dogs include static—intraarticular or extraarticular stabilization—and dynamic or tibial osteotomy techniques [6].

Tibial plateau leveling osteotomy (TPLO) and tibial tuberosity advancement (TTA)—both based on tibial osteotomies—are the most common surgical methods to provide dynamic stability during weight bearing by altering the geometry of the stifle joint [7, 8]. TPLO treats CCL rupture by neutralizing the cranial tibial thrust through an osteotomy rotated and fixated at a new plateau angle [9]. TTA—rooted on the Maquet procedure [10]—is based on the theory that the angle between the tibia plateau and the patellar ligament influences the production of tibio-femoral shear forces during limb loading [11]. Changing the angle between the patellar ligament and the tibial alignment to 90° shifts forces and eliminates stifle thrust [12, 13] providing good clinical outcomes [14]. Thus, performing the mediolateral osteotomy and advancing the tibial tuberosity cranially renders dynamic stability to the stifle joint [13, 15].

Experimental in vitro studies [16] and clinical information have proved the biomechanics and satisfactory limb function after TTA surgery [14]. In the conventional TTA technique description, after osteotomy the fixation was by forks and titanium cages of different sizes—from 3 to 12 mm [14, 16].

Other variations of TTA have been described and are currently been used. In 2014 the TTA Rapid technique-based on the Maquet technique [7]—was reported as a surgical method for dogs with CCR where the distal cortex of the tibial crest remains intact [17]. This variant of TTA is an alternative treatment for CCL rupture with good short- and medium-term outcomes, and low rate of complication [18, 19].

Studies support the hypothesis that the conformation of the tibial tuberosity has an influence on the advancement in TTA surgery, and the cage size and position relative to the tibial tuberosity also matters [20]. TTA-2 is a simplified variation of TTA that consists in a Maquet-like osteotomy, fixated with a new cage that eliminates stress risers created by the plate, fork, and screws, rendering plate and fork fixation unnecessary [21]. Stress risers were proven to occur in the osteotomized piece of bone as the holes are drilled perpendicular in the mediolateral plane for screw and fork placement [22].

Tension band wiring (TBW) technique is one of the most common surgical method used to treat a tension fracture [23–27]. TBW involves the use of two K-wires (1.1–2 mm) placed parallel to each other and a cranial anterior tension band of stainless steel wire in a vertically oriented figure-of-8 pattern [28], resulting in neutralization of tensile forces.

There is no study in the literature that has compared TBW with TTA for CCL rupture. To further clarify the role and effect of concurrent application of wire on the treatment outcomes, we designed a study comparing different used techniques (variations of TTA) with TBW to surgically treat CCL rupture. To the best of our knowledge, this is the first report to determine the impact of concurrently applying pins and wire as part of a TTA procedure on the treatment of CCL rupture.

TBW technique has been a well-documented surgical method. The role of concurrently applying a wire band on the tension surface to fractured patella to the osteotomy repair fixation is intended to help fracture stabilization. The purpose of this study was therefore to determine the impact of concurrent application of wire loop fixated on the tensile plane of the tibial tuberosity to stabilize the stifle joint.

## Methods

**Fig. 1:**
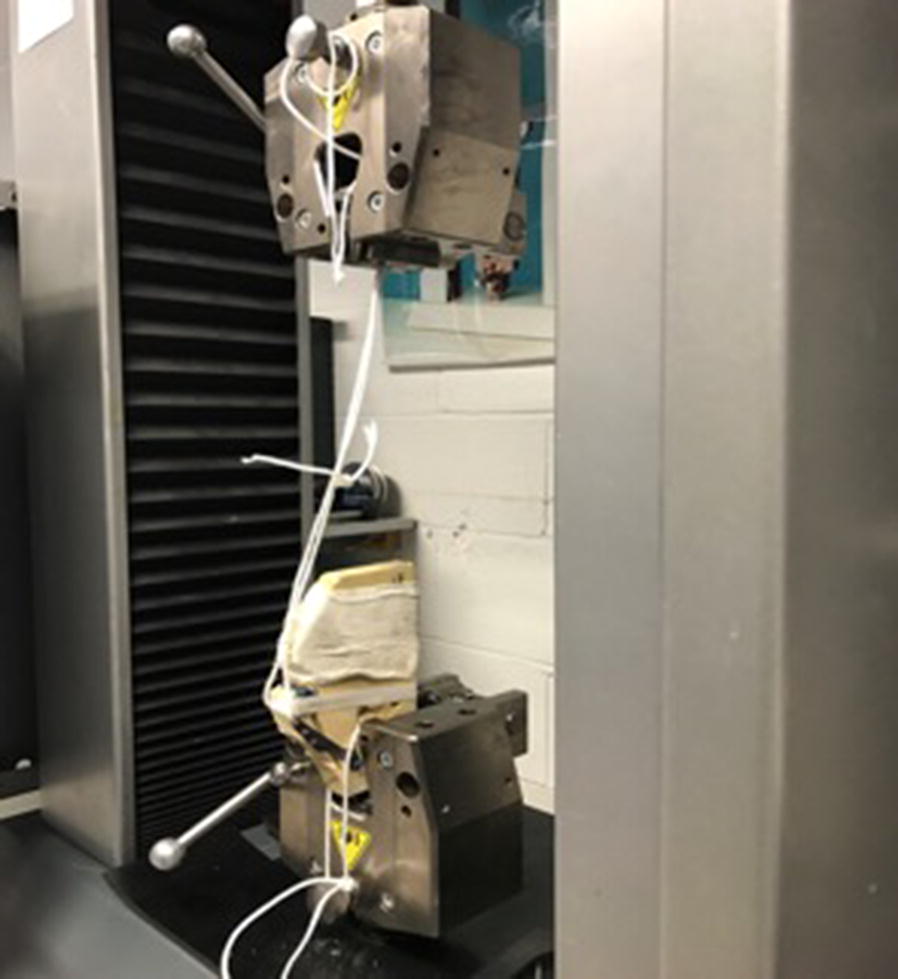
Image of the tensile testing machine used to measure tensile strenght in the experimental samples

An experimental study with artificial composite bone analogues (Sawbones^®^, Uppsala, Sweden)—validated to simulate human bone characteristics for fracture toughness, tensile strength, compressive strength, fatigue crack resistance and implant subsidence—was designed and performed to examine how the maximum failure strength varied between five different types of surgical fixation for CCR repair. Each sample was placed in a jig with a simulated quadriceps force (Fig. 1). To simulate the tibial tuberosity fixation a block of artificial bone was cut as if the fixation was a Macquet style repair, however the imitate the ventral fixation a 3.5 mm screw was inserted from the tuberosity into the main block.

### Design

**Fig. 2:**
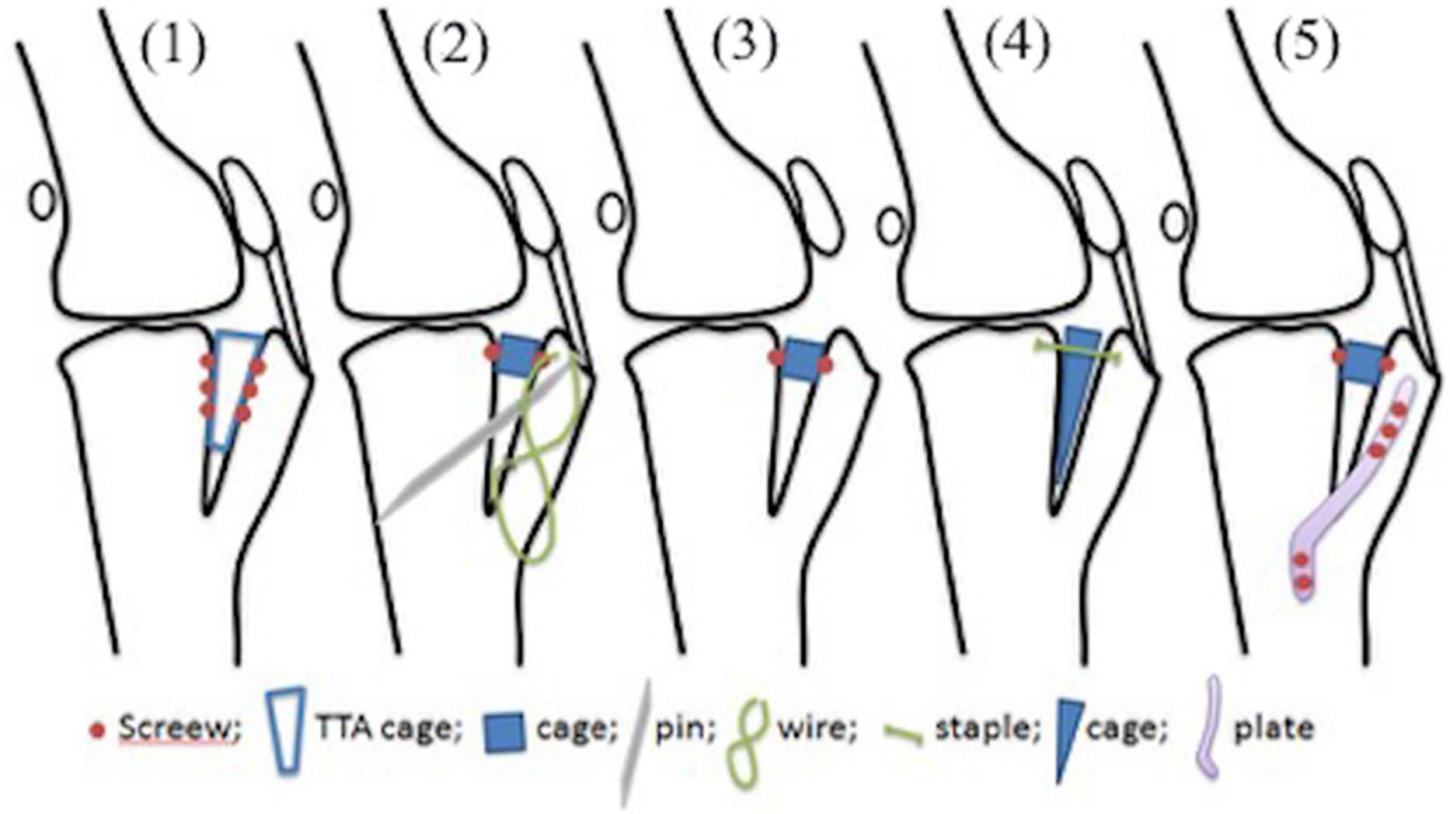
Pattern of suture used. Pictures show different surgical procedures: **a** TTA rapid; **b** TBW; **c** TTA cage; **d** TTA-2; **e** TTA plate

For testing, 8 samples were randomly assigned to 5 different groups: (1) TTA rapid group: osteotomy was held with cage and 6 screws [17]; (2) TBW group: osteotomy was held with pins and wire and cage and 2 screws [29]; 2 pins placed dorsal to cage, 1 screw caudal in the cage and one cerclage wire over pins on cranial aspect; cage is more dorsal and is at the top of the osteotomy (easy to remove then) and pins are ventral to cage; the pins do not interfere with the cage at all; (3) TTA cage group: osteotomy was held by cage and 2 screws only attached to the bone [30]; (4) TTA-2 group: osteotomy held by modified cage and staple [31]; and (5) TTA plate group: with cage and 2 screws, and plate and 5 screws [32]; this was used as control group. The different surgical techniques are shown in Fig. 2.

All cages used for the experimental design were the same (width—9 mm), and all screw in cage holes were 2 mm. The TTA plate was fixed with 2.7 mm screws to the main fragment.

The Sawbone blocks were cut to simulate a Maquet-like osteotomy with the distal part of the osteotomy fixated by a 3.5 mm screw. The elasticity of the artificial bone did not allow for retained fixation at the ventral aspect therefore a screw was placed to imitate this. Pins were drilled at an oblique angle into the cranial fragment just below the cage which was positioned more dorsally than would normally be. Distal hole for the passage of the wire was drilled ventral to the osteotomy line end 2 mm caudally into the cranial edge of the tibia.

The five groups of samples treated with different surgical technique were prepared for biomechanical analysis. Application of each of the TTA systems was carried out in line with manufacturer’s instructions. The samples were then fixed in a jig and tested to single cycle to fail in an Instron materials testing machine (Fig. 1) to test strength and resistance of the fixation technique using 1.5 N preload and test speed of 50 mm/min. All samples that underwent the biomechanical laboratory test were positioned in the testing jig and subjected to continuous increasing tension until the failure of fixation occurred in the sample using tension testing equipment. Maximum load at failure (N)—or strength of the fixation—was evaluated for each of the surgical technique used and compared statistically.

### Statistical methods

Statistical analysis was used to compare the strength of the five fixation types. Due to the continuous nature of the outcome variable, the values were compared between the five types of surgical fixations using Analysis of Variance (ANOVA). In addition to the overall comparison between the five groups, post hoc tests were used to compare between pairs of fixations. Since multiple comparisons for each fixation were executed, a Bonferroni adjustment was used to inflate the P-values in order to allow for multiple testing. Judging distribution in small sample sizes was by graphical and visible inspection to look for any non-normal patterns.

Models were implemented in commercially available software (IBM SPSS Statistics Version 23, International Business Machines Corp., Armonk, NY, USA) and results were considered to be of statistical significance if P-value < 0.05.

## Results

TBW presented the higher resistance to failure when loaded, compared to all the other surgical procedures, while the other techniques have a similar behaviour in pairs (TTA rapid and TTA plate were alike, while TTA cage and TTA-2 displayed similar strength). The highest values were found in the TBW group with 1.47 ± 0.07 N, with the lowest mean value in the TTA cage and TTA2 groups. The differences were statistically significant in all cases with P < 0.001, suggesting that a highly significant overall difference in strength between the five groups. Table 1 shows the results from the experiment comparing the different surgical technique used. Data is expressed as the mean and standard deviation strength values in each group.

**Table 1:** Strength values of the groups treated with different surgical techniques used

Fixation method	n	Strength Mean ± SD	Overall P-value
TTA rapid	8	1.31 ± 0.07**^#^	< 0.001
TBW	8	1.47 ± 0.06^^^	
TTA cage	8	0.82 ± 0.08**^##^	
TTA-2	8	0.85 ± 0.06**^##^	
TTA plate	8	1.26 ± 0.06**^#^	

Results expressed as mean ± standard deviation; **P < 0.001; ^#^similar group behaviour (TTA rapid and TTA plate); ^##^similar group behaviour (TTA cage and TTA-2); ^**^**^different behavior to all other groups

Four groups exhibited a comparable behaviour by pairs—TTA rapid and TTA plate performed in a similar way, while TTA cage and TTA-2 also acted alike—TBW displayed a completely different pattern of conduct.

**Fig. 3:**
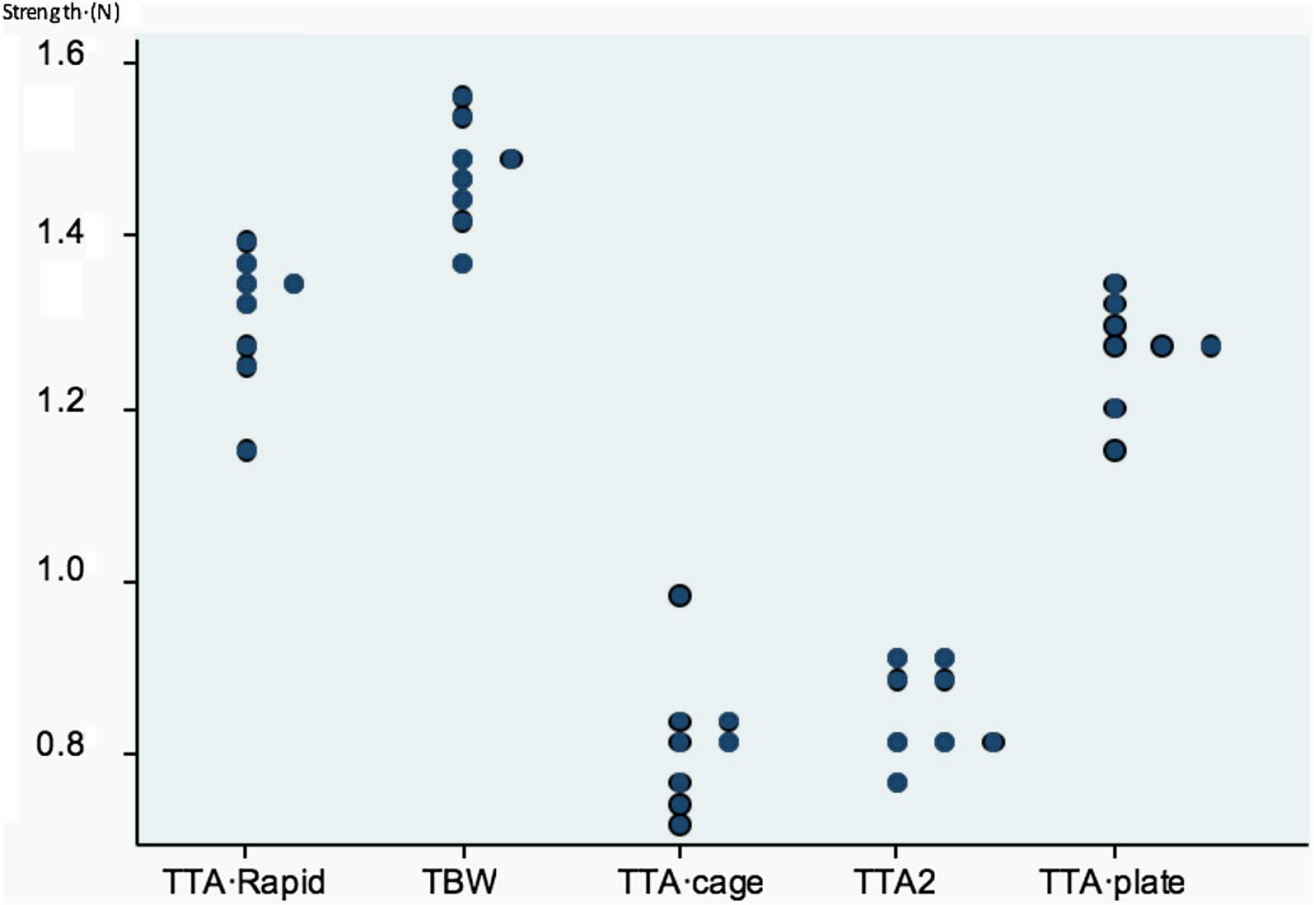
Dotplot representing the distribution of the samples treated with each surgical technique at failure

Further pair-wise comparisons were performed to examine the difference between specific groups. These analyses found no significant differences between the TTA rapid and TTA plate groups, or between the TTA-2 and TTA cage groups (both with P = 1.00). However, all other pair of groups were significantly different with P < 0.001. A graphical illustration of the results in the five groups is shown in Fig. 3.

The properties of artificial bone are manufactured to be similar to bone but it is only an approximation. Also achieving the exact shape of the real proximal tibia was not possible therefore the shape is also an approximation. These factors do not allow for the different breeds, shapes and sizes of real tibias. However, the positive aspect of using artificial bone is that it is consistent in its properties and best for comparative tests such as the study performed here.

## Discussion

Our study showed that TBW provides greater strength than any of the other techniques used for treating CCL rupture when biomechanically tested to failure. Tension band wiring (TBW) in combination with a plate has been traditionally used in arthrodesis and is also employed in osteosynthesis [33] and symphysis procedures [34]. However, TBW is not a technique typically used in CCL rupture. TTA plate—used as control—displayed 16.66% less strength than the TBW technique and 3.96% less than TTA rapid sampled, but exhibited more resistance than TTA cage and TTA-2 (34.93% and 32.54%, respectively). However, when compared to TBW, all the other techniques decreased substantially demonstrated that they weaker fixation when tested to failed (TTA rapid: 10.89%; TTA cage: 44.22%; TTA-2: 42.18%; and TTA plate 14.29%). To our knowledge, this is the first time that TTA and its variations has been compared to TBW as a surgical procedure to repair CCL.

Regarding the tibial osteotomy techniques, subjectively, TTA appears to be a useful alternative in the management of CCL rupture and can effectively change the degree and direction of the tibiofemoral shear force, counteracting cranial subluxation of the tibia during simulated weight-bearing in biomechanical studies [13], and providing stability in CCL-deficient stifle joint [15].

Experimental and retrospective studies showed that TTA significantly improved the lameness, activity level [35], and limb function in dogs with CCL disease [36], but did not result in complete return to function, and only approximately 90% of normal can be expected in dogs with CCL disease undergoing TTA. Experimental biomechanical studies showed that TTA restored the normal femorotibial joint and patellofemoral alignment, and reduced the retropatellar force [37], the patellar tendon load, and the cranial tibial thrust in the CCL-deficient stifle joint through an alteration of tibial plateau angle in CCL deficient stifles of dogs [38].

Skinner et al. [39] assessed femorotibial joint alignment under static weight-bearing conditions in dogs treated for CCL insufficiency with TTA, and although TTA did not normalize sagittal plane femorotibial stability, most dogs returned to good limb function regardless of femorotibial alignment.

Variables that influence the outcome of TTA include high body weight and preoperative patellar tendon angle [40]. Although some complications adversely affected functional outcome [19], in a retrospective study, Steinberg et al. [41] concluded that TTA had a complication rate and owner satisfaction similar to other tibial osteotomies for the surgical correction of CCL disease. Complications encountered included implant failure, tibial crest displacement and medial meniscal tears [40, 42, 43].

The occurrence of intraoperative fracture during advancement of an elongated bi-directional hinged osteotomy for TTA in dogs of the tibial tuberosity is less when a hemicerclage suture is placed [44]. The tendency to failure is because the traditional fixation methods rely on mediolateral fixation [22] whereas the TBW is situated on the cranial plane, and therefore in line with the pull of the quadriceps.

Edwards et al. [45] reported major complications in a large cohort of dogs following TTA using either a fork-based or a screw-based implant system. Amongst the variables evaluated that might have influenced this result were the implant type and body weight. The incidence of complications associated with TTA surgical procedures—including tibial tuberosity fractures—appear to be predominant in certain dog breed such as Boxer, as shown in a retrospective study.

Skytte et al. [46] showed that an early treatment of partially ruptured CCL with a TPLO or TTA provides a better long-term result with the remaining CCL staying intact in most cases providing the affected stifle with stability. Major complications occurred frequently following TTA and TPLO treatment of CCL disease in heavier dogs (over 50 kg).

Our study suggests that fixating the osteotomy with a TBW technique would increase the strength of the fixation and decreased complication rate of CCL rupture repair compared to other TTA surgical procedures, especially in a scenario of extra loading. This is because the rate of implant failure would be reduced and would be particularly suitable for large or overweight dogs or bilateral cases where loading would be very high. Further in vivo studies are necessary to confirm these results and incorporate TBW as a preferred surgical technique to treat CCL rupture.

Korvic et al. [47] showed that the patellar force in goats weighing approximately 60 kg during standing in place was 207 N and 1000 N during a trot. The force to failure of the goat patella tendon is 3100 N. In comparison to our results the force to failure was at its highest at 1.47 N which although not near 3100 N it is closer to the normal loading force at 1000 N. To our knowledge, there is no such study in dogs, so it is difficult to extrapolate to real values. In addition, we are using an artificial model, and therefore, a limitation of the study would be the possibility that the forces acting on the tibial tuberosity might be higher than those calculated with our device. However, our purpose was to compare different surgical techniques for CCL repair and find out was the strongest, and that purpose was achieved, since all the samples underwent the same experimental tensional force. The main limitation of the study is that we are using artificial bone, but we think that its consistency outweighs the limit. Other limitations are related to the forces generated and the lack of patellar tendon insertion.

## Conclusions

The results of the experiment on CCL rupture in an artificial bone model revealed that TBW procedure was the most resilient surgical fixation for CCL repair presenting the higher strength when tested to failure. Orienting the fixation onto the cranial tensile plane optimizes the fixation behavior. Further clinical studies are needed to demonstrate in vivo reliability and to test different variables such as size and weight of dogs.

## Data Availability

The datasets used and/or analyzed in the current study are available from the corresponding author on reasonable request.

## Abbreviations

CCL: cranial cruciate ligament (proper terminology in dogs)
TBW: tension band wire
TPLO: tibial plateau leveling osteotomy
TTA: tibial tuberosity advancement
